# Endometrial clear cell carcinoma: A population-based study

**DOI:** 10.3389/fonc.2022.961155

**Published:** 2022-10-24

**Authors:** Pengfei Cui, Xiaofeng Cong, Youhao Zhang, Huimin Zhang, Ziling Liu

**Affiliations:** Cancer Center, The First Hospital of Jilin University, Changchun, China

**Keywords:** endometrial clear cell carcinoma, prognosis, nomogram, FIGO stage, risk classification system

## Abstract

**Background:**

A systematic analysis of prognostic factors concerning endometrial clear cell carcinoma (ECCC) is lacking. The current study aimed to construct nomograms predicting the overall survival (OS) of ECCC patients.

**Methods:**

We performed a retrospective study, and predicted nomograms for 3-, 5-, and 10-year OS were established. The nomograms were verified with the consistency index (C-index), calibration curve, and decision curve analysis (DCA).

**Results:**

A total of 1778 ECCC patients, 991 from FIGO stage I/II and 787 from FIGO stage III/IV, were included in this study. The age at diagnosis, marital status, T stage, tumor size, and surgery-independent prognostic factors in FIGO stage I/II, and the age at diagnosis, T stage, lymph node involvement, distant metastasis, tumor size, surgery, radiotherapy, and chemotherapy in FIGO stage III/IV were independent prognostic factors. The C-indexes of the training and validation group were 0.766 and 0.697 for FIGO stage I/II and 0.721 and 0.708 for FIGO stage III/IV, respectively. The calibration curve revealed good agreement between nomogram-predicted and actual observation values. The DCA established that nomograms had better clinical benefits than the traditional FIGO stage.

**Conclusions:**

The predicted nomograms showed good accuracy, excellent discrimination ability, and clinical benefits, depicting their usage in clinical practice.

## Introduction

Endometrial carcinoma (EC) is the sixth most diagnosed cancer among women (1). An estimated 66,570 new cases of uterine corpus cancer and 12,940 deaths were reported in the United States in 2021 (2). EC is usually diagnosed during stage I, and patients have a good prognosis as it induces symptoms from an early stage (3). Postmenopausal vaginal bleeding is the most common symptom of EC (3, 4). Additionally, tumor invasion of the cervix can lead to blood or pus in the uterine cavity, causing abdominal swelling and cramping pain. Patients having advanced disease may suffer pelvic and lumbosacral pain due to the invasion of the tumor within the surrounding tissues or nerves (3, 4). EC is classified as type I and type II based on Bokhman’s dualist model^5^. Type I EC is estrogen-dependent and accounts for nearly 80% of all EC. Its pathological type is primarily endometrioid carcinoma (3–5). Endometrial clear cell carcinoma (ECCC) is a type II EC accounting for approximately 2–4% of the total EC, and is more common in older women (6). ECCC is an estrogen-independent tumor whose onset has no apparent relationship with estrogen (4, 5, 7). ECCC is more aggressive and prone to early metastasis than endometrioid carcinoma (4, 5, 7). Many studies report a 5-year survival rate of less than 50%, irrespective of the ECCC clinical stage. However, all these studies included small samples having limited persuasion (4). Only a few small retrospective cohort studies and some case reports have explored the prognostic factors in ECCC due to its low incidence. Notably, there are no systematic analyses of ECCC from a large population sample. Therefore, the current study aimed to perform a comprehensive retrospective analysis depending on the Surveillance, Epidemiology, and End Results (SEER) database to evaluate the survival and prognostic risk factors for ECCC. Moreover, it establishes definitive individualized prognostic prediction models to predict the 3-, 5- and 10-year overall survival (OS) in ECCC patients. The findings contribute to developing appropriate treatment and follow-up strategies for ECCC.

## Methods

### Data source and patient selection

The present study recruited ECCC patients from 18 registries of the SEER database between 2000 and 2018 using the SEER* Stat software (version 8.3.9). The National Cancer Institute established the SEER database in 1973, covering approximately 28% of the U.S. population. It includes age, sex, race, and year of diagnosis (8). All the data for this study were retrieved from the SEER database.

The inclusion criteria for this study were: (1) Primary site-labeled: C54.1-Endometrium. (2) ICD-O-3 Hist/behave: 8310/2: Clear cell adenocarcinoma *in situ*, 8310/3: Clear cell adenocarcinoma, NOS. (3) Year of diagnosis: 2000-2018. (4) Diagnosis confirmed based on histology or cytology. (5) Single primary cancer.

The exclusion criteria were: (1) The survival time was 0 and unknown. (2) The T stage was T0. (3) Unknown race. (4) Unknown AJCC stage.

### Variable selection

Each of the following variables was considered for every patient: age at diagnosis, marital status (married, divorced, separated, unmarried, widowed, or unknown), race (white, black, or other), T stage (T1, T2, T3, T4, or TX), lymph node involvement (no, yes, or unknown), distant metastasis (no, yes, or unknown), tumor size (< 4.5 cm, 4.5–6.1 cm, > 6.1 cm or unknown), grade (I: well differentiated, II: moderately differentiated, III: poorly differentiated, IV: undifferentiated, or unknown), the International Federation of Gynecology and Obstetrics (FIGO) stage (I, II, III, or IV), surgery (partial hysterectomy, or total hysterectomy), radiotherapy (no or yes), chemotherapy (no/unknown, or yes), vital status (dead or alive), and time of survival (length in months). The FIGO stage of the patients was obtained based on the TNM staging system since no data on the FIGO stage was available in the SEER database. The endpoint of this study was OS, defined as the time from diagnosis to death or from the last follow-up (patients lost to follow-up).

### Statistical analysis

The Chi-square test and Cox regression analysis were performed with the SPSS software. In contrast, the R software performed the C-index, calibration curve, DCA, and Log-rank tests. The Chi-square test determined the potential statistical differences in the demographic clinicopathological features and treatment patterns among patients with early and advanced ECCC. Then, patients having ECCC in FIGO stages I/II and III/IV were randomly assigned to the training and validation cohort at a 7:3 ratio, respectively. Univariate and multivariate Cox regression analyses were performed in the FIGO I/II and III/IV training cohorts to identify the independent OS risk factors. Predictive nomograms were established depending on the results of the Cox regression analysis for OS to predict the 3-, 5- and 10-year OS. The accuracy of the nomograms was validated with the consistency index (C-index). Moreover, the calibration curves were developed to compare the consistency between the OS predicted by the nomogram at 3, 5, and 10 years and their actual values. The clinical benefit of the nomograms and classical FIGO staging system was compared through a decision curve analysis (DCA). The survival curve of patients from different risk groups was analyzed with the Log-rank tests. The significance threshold had been set at *P* < 0.05.

## Results

### Demographics and clinical characteristics

A total of 1,778 patients diagnosed with ECCC were enrolled in this study between 2000 and 2018, depending on the inclusion and exclusion criteria. They were divided into 991 (55.7%) patients from FIGO stage I/II and 787 (44.3%) from FIGO stage III/IV. The demographic features of patients with ECCC are listed in **Table 1**. The median age of the patients was 68 years. Most patients were white (72.5%) and had been subjected to total hysterectomy (87.3%). Almost one-third of the patients (29.3%) had a tumor < 4.5 cm in size, 45.7% were married, 47.6% were in pathological grade III, 42.5% received radiotherapy, and 46.2% received chemotherapy. There were statistical differences between the FIGO stage I/II and FIGO Stage III/IV patients in marital status, race, tumor size, grade, surgery, radiotherapy, and chemotherapy (all *P* < 0.05). The number of patients with tumor size < 4.5 cm and pathological grade I in FIGO stage I/II was more than that in FIGO stage III/IV (36.2% vs. 20.6% and 1.9% vs. 0.8%, respectively) respectively). In contrast, the number of patients with tumor size > 6.1cm and pathological grade III in FIGO Stage III/IV was more than that in FIGO stage I/II (23.1% *vs.* 8.2% and 51.1% *vs.* 44.9%, respectively). Total hysterectomy and radiotherapy rates in the FIGO stage I/II were 92.1% and 46.9%, respectively, higher than the FIGO stage III/IV (81.2% and 36.8%).

**Table 1:** Basic characteristics of ECCC patients from the total population, FIGO stage I/II, and FIGO stage III/IV cohorts.

Variables	Total population		FIGO stage I/II		FIGO stage III/IV		P value
	N=1778		N=991		N=787		
**Age (years)**	68 (27~96)		68 (31~96)		68 (27~95)		0.165
**Marital status**							0.018
Married	813	45.7	479	48.3	334	42.4	
Divorced	192	10.8	99	10.0	93	11.8	
Separated	22	1.2	11	1.1	11	1.4	
Unmarried	275	15.5	133	13.4	142	18.0	
Widowed	392	22.0	215	21.7	177	22.5	
Unknown	84	4.7	54	5.4	30	3.8	
**Race**							0.002
White	1289	72.5	739	74.6	550	69.9	
Black	312	17.5	146	14.7	166	21.1	
Other	177	10.0	106	10.7	71	9.0	
**Tumor size (cm)**							<0.001
<4.5	521	29.3	359	36.2	162	20.6	
4.5-6.1	226	12.7	106	10.7	120	15.2	
>6.1	263	14.8	81	8.2	182	23.1	
Unknown	768	43.2	445	44.9	323	41.0	
**Grade**							0.006
I	25	1.4	19	1.9	6	0.8	
II	95	5.3	65	6.6	30	3.8	
III	847	47.6	445	44.9	402	51.1	
IV	331	18.6	189	19.1	142	18.0	
Unknown	480	27.0	273	27.5	207	26.3	
**Surgery**							<0.001
Partial hysterectomy	226	12.7	78	7.9	148	18.8	
Total hysterectomy	1552	87.3	913	92.1	639	81.2	
**Radiotherapy**							<0.001
No/Unknown	1023	57.5	526	53.1	497	63.2	
Yes	755	42.5	465	46.9	290	36.8	
**Chemotherapy**							<0.001
No/Unknown	957	53.8	697	70.3	260	33.0	
Yes	821	46.2	294	29.7	527	67.0	
**Median OS (m)**	34		56 (1-227)		21 (1-225)		<0.001
**Cases of dead**	926		366	39.5	560	60.5	

### Independent risk factors for OS

Univariate and multivariate cox analyses indicated that age at diagnosis, marital status, T stage, tumor size, and surgery were independent risk factors for OS among patients with FIGO stage I/II (all *P* < 0.05) (**Table 2**). Additionally, age at diagnosis, T stage, lymph node involvement, distant metastasis, tumor size, surgery, radiotherapy, and chemotherapy were independent predictors for OS stage III/IV patients (*P* < 0.05) (**Table 3**).

**Table 2:** Univariate and multivariate COX analyses of OS in the FIGO stage I/II training cohort.

Variables	Univariate analysis				Multivariate analysis			
	HR	95%CI		P value	HR	95%CI		P value
		Lower	Upper			Lower	Upper	
**Age (years)**	1.078	1.064	1.091	<0.001	1.071	1.056	1.086	<0.001
**Marital status**								
Divorced vs Married	1.450	0.933	2.252	0.098	1.183	0.758	1.846	0.460
Separated vs Married	1.513	0.478	4.786	0.481	3.892	1.194	12.687	0.024
Unmarried vs Married	1.170	0.772	1.772	0.460	1.423	0.922	2.198	0.111
Widowed vs Married	2.954	2.204	3.959	<0.001	1.453	1.054	2.003	0.023
Unknown vs Married	1.479	0.828	2.640	0.186	1.031	0.572	1.856	0.920
**Race**								
Black vs White	1.055	0.748	1.489	0.759	0.713	0.494	1.028	0.070
Other vs White	0.558	0.348	0.894	0.015	0.795	0.492	1.285	0.349
**T**								
T2 vs T1	2.029	1.553	2.650	<0.001	1.700	1.283	2.252	<0.001
**Tumor size (cm)**								
4.5~6.1 vs <4.5	0.739	0.428	1.274	0.276	0.632	0.364	1.096	0.102
>6.1 vs <4.5	1.998	1.244	3.211	0.004	1.884	1.140	3.112	0.013
Unknown vs <4.5	1.434	1.070	1.922	0.016	1.261	0.926	1.716	0.141
**Grade**								
II vs I	1.511	0.567	4.028	0.409	–	–	–	–
III vs I	1.564	0.639	3.829	0.328	–	–	–	–
IV vs I	1.385	0.548	3.501	0.491	–	–	–	–
Unknown vs I	1.456	0.586	3.621	0.419	–	–	–	–
**Surgery**								
Total hysterectomy vs Partial hysterectomy	0.175	0.126	0.243	<0.001	0.230	0.158	0.336	<0.001
**Radiotherapy**								
Yes vs No/Unknown	1.062	0.829	1.360	0.633	–	–	–	–
**Chemotherapy**								
Yes vs No/Unknown	0.692	0.507	0.945	0.021	1.147	0.824	1.598	0.417

**Table 3:** Univariate and multivariate COX analyses of OS in the FIGO stage III/IV training cohort.

Variables	Univariate analysis				Multivariate analysis			
	HR	95%CI		P value	HR	95%CI		P value
		Lower	Upper			Lower	Upper	
**Age (years)**	1.028	1.017	1.038	<0.001	1.020	1.008	1.032	0.001
**Marital status**								
Divorced vs Married	1.472	1.050	2.062	0.025	1.097	0.775	1.553	0.603
Separated vs Married	1.508	0.618	3.679	0.367	1.245	0.500	3.101	0.637
Unmarried vs Married	1.232	0.928	1.636	0.149	1.071	0.794	1.444	0.655
Widowed vs Married	1.831	1.427	2.350	<0.001	1.172	0.889	1.544	0.261
Unknown vs Married	0.896	0.518	1.550	0.694	0.598	0.338	1.060	0.079
**Race**								
Black vs White	1.444	1.130	1.845	0.003	1.275	0.986	1.649	0.064
Other vs White	0.828	0.574	1.194	0.311	0.866	0.595	1.260	0.452
**T stage**								
T2 vs T1	1.376	0.898	2.108	0.142	1.288	0.830	1.997	0.259
T3 vs T1	1.886	1.365	2.606	<0.001	2.037	1.452	2.858	<0.001
T4 vs T1	2.984	1.992	4.469	<0.001	2.297	1.492	3.536	<0.001
TX vs T1	3.543	2.364	5.309	<0.001	1.880	1.188	2.974	0.007
**Lymph nodes involvement**								
Yes vs No	0.905	0.729	1.124	0.367	1.430	1.129	1.812	0.003
Unknown vs No	1.925	1.417	2.617	<0.001	1.476	1.052	2.070	0.024
**Distant metastasis**								
Yes vs No	2.441	1.993	2.989	<0.001	1.970	1.538	2.524	<0.001
Unknown vs No	5.839	0.813	41.928	0.079	1.325	0.173	10.153	0.787
**Tumor size (cm)**								
4.5~6.1 vs <4.5	1.465	1.037	2.068	0.030	1.482	1.039	2.114	0.030
>6.1 vs <4.5	1.275	0.924	1.759	0.140	1.180	0.842	1.655	0.337
Unknown vs <4.5	1.596	1.208	2.109	0.001	1.053	0.763	1.452	0.754
**Grade**								
II vs I	0.71	0.226	2.233	0.558	–	–	–	–
III vs I	0.897	0.333	2.414	0.830	–	–	–	–
IV vs I	0.753	0.274	2.069	0.583	–	–	–	–
Unknown vs I	0.888	0.327	2.414	0.816	–	–	–	–
**Surgery**								
Total hysterectomy vs Partial hysterectomy	0.337	0.264	0.430	<0.001	0.372	0.283	0.490	<0.001
**Radiotherapy**								
Yes vs No/Unknown	0.550	0.444	0.682	<0.001	0.680	0.537	0.861	0.001
**Chemotherapy**								
Yes vs No/Unknown	0.568	0.462	0.698	<0.001	0.582	0.461	0.734	<0.001

### Nomograms for the prediction of OS

**Figure 1:**
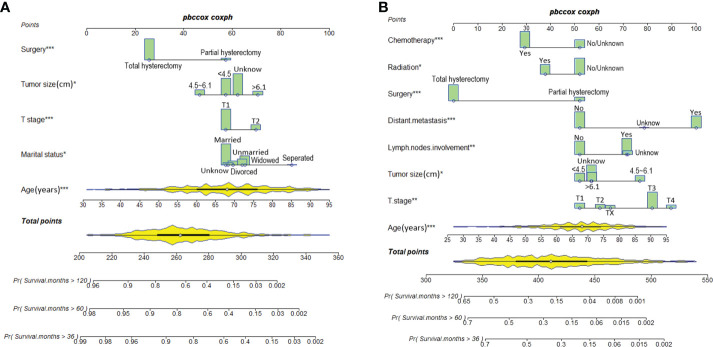
Nomograms for predicting 3-, 5-, and 10-year OS among patients with FIGO stage I/II **(A)** and FIGO stage III/IV **(B)** ECCC.

**Figure 2:**
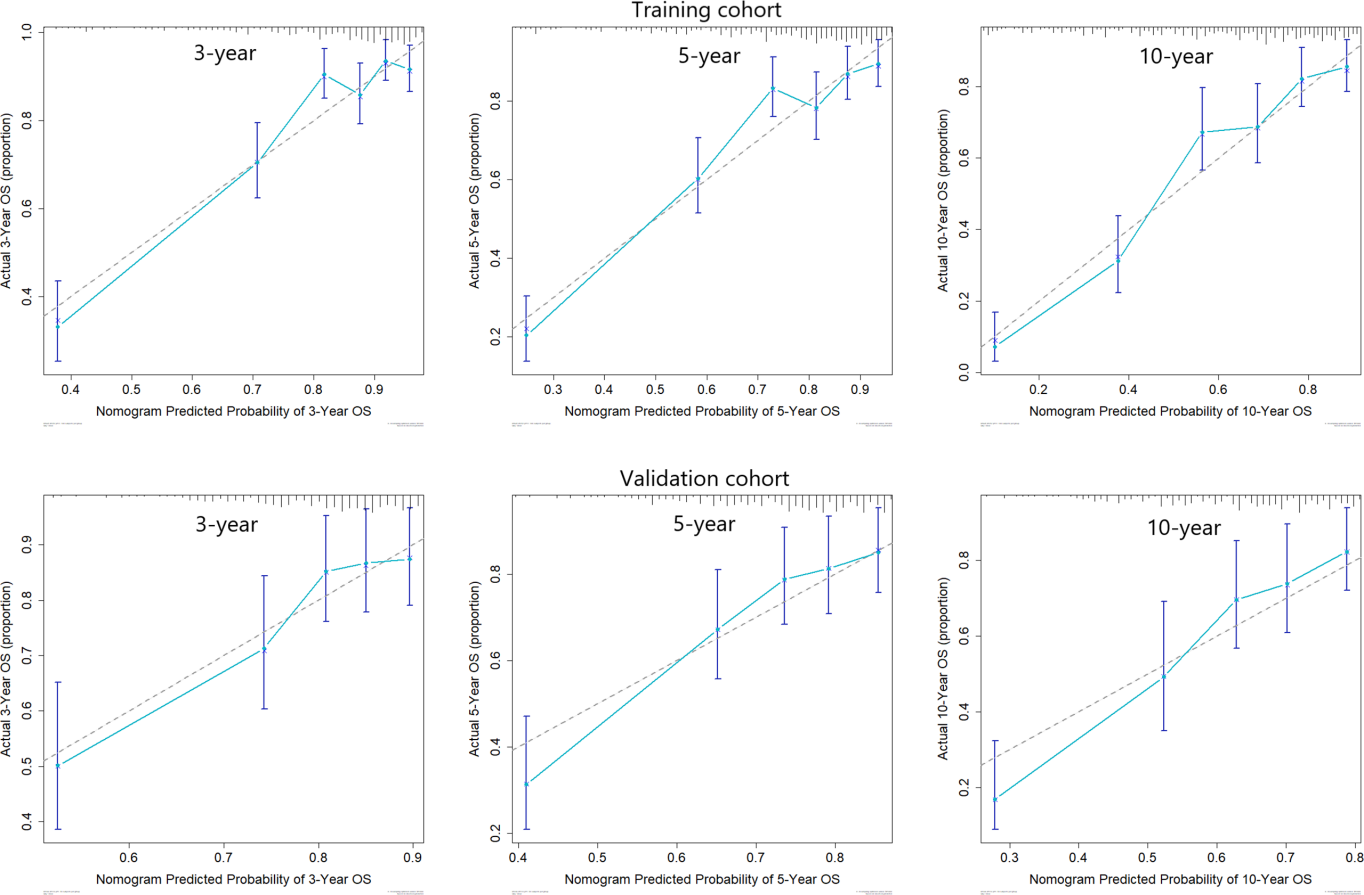
Calibration curves for 3-, 5-, and 10-year OS among patients with FIGO stage I/II ECCC within the training and validation cohorts.

**Figure 3:**
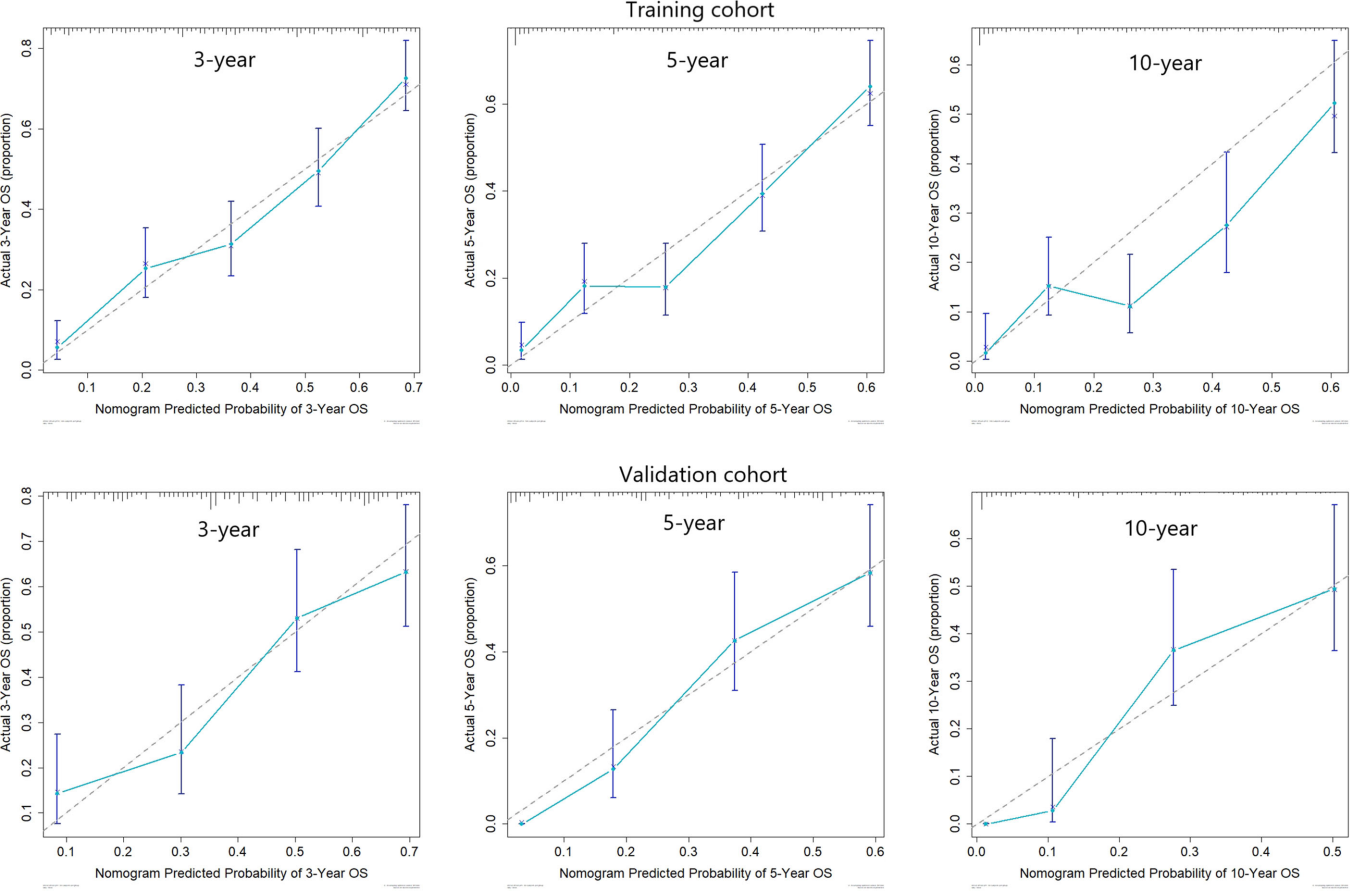
Calibration curves for 3-, 5-, and 10-year OS among patients with FIGO stage III/IV ECCC within the training and validation cohorts.

**Figure 4:**
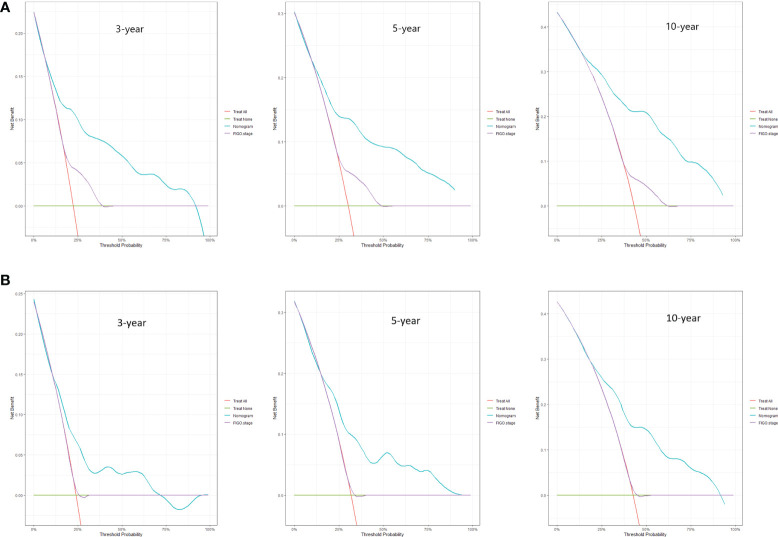
DCA curves for 3-, 5-, and 10-year OS among ECCC patients with FIGO stage I/II within the training cohort **(A)** and validation cohort **(B)**.

**Figure 5:**
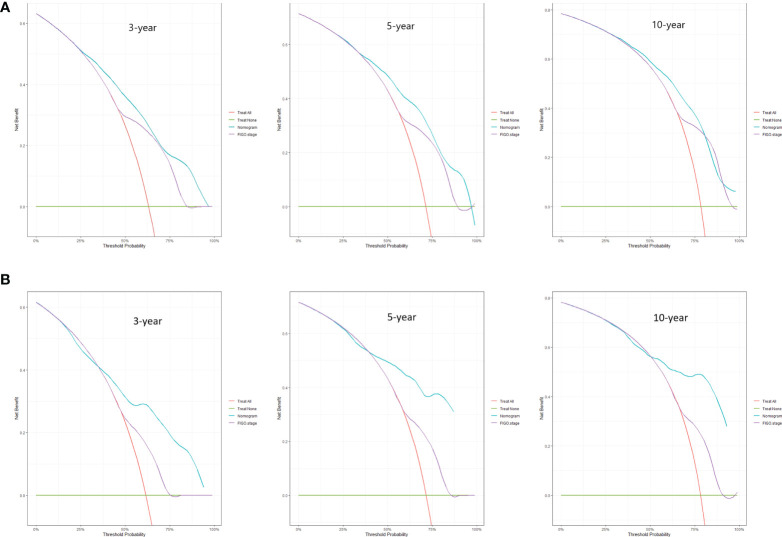
DCA curves for 3-, 5-, and 10-year OS among ECCC patients with FIGO stage III/IV within the training cohort **(A)** and validation cohort **(B)**.

Predictive nomograms were developed depending on the independent risk variables to predict the 3-, 5- and 10-year OS (**Figure 1**). Surgery (31 scores) had the most prognostic impact on FIGO stage I/II among the categorical variables. It was followed by marital status (28 scores), tumor size (24 scores), and T stage (13 scores) (**Table 4**). Surgery (52 scores) was also the most important factor in the FIGO stage III/IV among the categorical variables, followed by distant metastasis (48 scores), T stage (38 scores), tumor size (25 scores), chemotherapy (23 scores), lymph node involvement (20 scores), and radiotherapy (14 scores) (**Table 5**). The internal and external validations of the nomograms were performed in the training and validation cohorts, respectively. The C-indexes of the training and validation groups from the FIGO stage I/II and FIGO stage III/IV were 0.766 (95% CI: 0.750-0.782) and 0.697 (95% CI: 0.640–0.754), and 0.721 (95% CI: 0.708–0.734) and 0.708 (95% CI: 0.667–0.749), respectively. These values depicted that the constructed nomograms showed a good predictive performance. In addition, the calibration curves of the two groups had a good agreement between the nomogram-predicted and the real observation values (**Figures 2** and **3**). The DCA models revealed that the nomograms outperformed the FIGO staging system in clinical benefit (**Figures 4** and **5**), suggesting that nomograms showed more predictive power than the traditional staging system.

**Table 4:** Nomogram scores of each independent prognostic factor in the FIGO stage I/II ECCC patients.

Variables	Scores
**Age (years)**	1.56*Age-48.71
**Marital status**	
Married	57
Divorced	60
Separated	85
Unmarried	64
Widowed	65
Unknown	58
**T stage**	
T1	57
T2	70
**Tumor size (cm)**	
<4.5	57
4.5~6.1	47
>6.1	71
Unknown	62
**Surgery**	
Partial hysterectomy	57
Total hysterectomy	26

**Table 5:** Nomogram scores of each independent prognostic factor in the FIGO stage III/IV ECCC patients.

Variables	Scores
**Age (years)**	1.29*age-34.59
**T stage**	
T1	52
T2	60
T3	82
T4	90
TX	65
**Lymph nodes involvement**	
No	52
Yes	71
Unknown	72
**Distant metastasis**	
No	52
Yes	100
Unknown	79
**Tumor size (cm)**	
<4.4	52
4.5~6.1	77
>6.1	57
Unknown	57
**Surgery**	
Partial hysterectomy	52
Total hysterectomy	0
**Radiotherapy**	
No/Unknown	52
Yes	38
**Chemotherapy**	
No/Unknown	52
Yes	29

### The novel risk-stratification system

**Figure 6:**
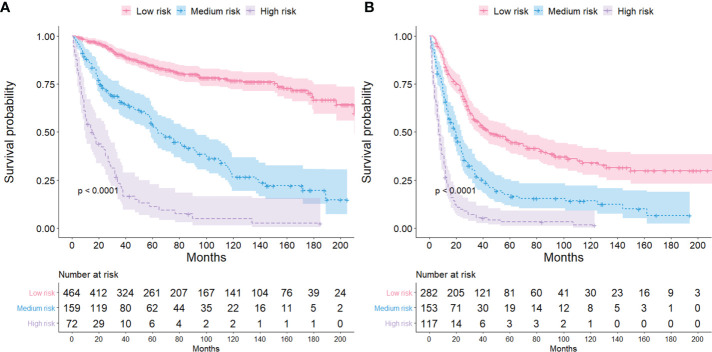
Kaplan-Meier survival curves for different risk groups among ECCC patients with FIGO stage I/II **(A)** and FIGO stage III/IV **(B)**.

The ECCC patients were divided into low-risk, medium-risk, and high-risk groups based on the total scores from each variable. The median survival time in the high, medium, and low-risk groups for the FIGO stage I/II were 14.5, 40, and 69 months (**Figure 6A**), respectively, and 7, 18, and 33 months, respectively (**Figure 6B**) for the FIGO stage III/IV. The log-rank tests revealed that the survival times for the three risk groups differed significantly (both *P* < 0.001).

## Discussion

Unlike EC, most ECCC patients have a tumor negative for the estrogen and progesterone receptors. However, positive for hepatocyte nuclear factor 1β and Napsin A. Notably, *TP53* is the most commonly mutated gene in ECCC (4, 9–11). The abnormal p53 expression is considered a poor prognostic factor for EC (11). Previous studies observed that the mutation rate of the *TP53* gene in POLE wild-type ECCC is 46%, while that of non-POLE endometrioid carcinoma is only 11% (11). ECCC patients are accompanied by high-risk factors for poor prognoses, including advanced clinical stage, deep muscular infiltration, lymphovascular space involvement, and distant metastasis, with a high recurrence rate, high mortality, and poor prognosis than in type I EC (3, 4, 7). Currently, The Cancer Genome Atlas (TCGA) classification is the most authoritative classification system of EC. However, it does not include ECCC patients. Therefore, it is essential to analyze the demographic and clinicopathological characteristics of ECCC patients independently. Moreover, we must comprehensively evaluate their prognosis to develop an adequate treatment guide for ECCC patients.

Our study identified that age was an essential prognostic factor among ECCC patients, positively correlating with the risk of death. The findings of this study concerning the relationship between age and prognosis in EC patients were consistent with previous studies. A retrospective study found that patients aged ≤ 40 include more favorable prognostic factors, such as a higher proportion of non-invasive carcinoma, a lower proportion in the uterine segment involvement, and less invasion of the lymphatic vascular space than in EC patients aged 40-60 years (12). Furthermore, EC patients aged ≤ 40 years had a lower probability of mismatch repair defects due to MLH1 methylation, a mutation associated with poor prognosis, than patients aged 41-60 years (12). Another study also found that ECCC patients aged ≥ 70 had worse progression-free survival time and OS independent of the treatment modality they were subjected to (13). An investigation on the influence of marital status on the diagnosis and prognosis of EC revealed that marriage was a protective prognostic factor for OS and cancer-specific survival among EC patients. Unmarried, divorced/separated, and widowed patients showed a higher risk of death than married patients (14). This phenomenon was because married patients were more likely to be diagnosed early, possibly due to the stability of the endogenous hormone levels in women associated with emotional benefits (14). In this study, separated and widowed patients having early ECCC had a higher risk of death than married patients at the same stage. However, marital status had no significant effect on the prognosis of patients with advanced ECCC. Previous studies have evaluated the relationship between tumor diameter, myometrium invasion depth, and prognosis of EC patients. Nilufer et al. observed that more than half of the ECCC patients with a tumor diameter > 2 cm were prone to myometrial invasion (15). Kohei et al. identified that large tumor size is associated with deeper myometrial infiltration and lymph node metastasis among EC patients (16). In this study, ECCC patients with large tumor sizes and late T stages significantly enhanced the risk of death. These findings were consistent with a retrospective study that inferred that large tumor size and deep muscle invasion could be associated with poor prognosis among ECCC patients (17). Lymphatic metastasis is the main route of EC metastasis. The survival time of patients is significantly shortened once they develop lymph node metastasis, indicating disease progression (18). This study also depicted that OS is significantly decreased in ECCC patients with lymph node involvement.

A study revealed that black patients with EC were more likely to develop invasive and non-endometrioid cancer than white EC patients from America (19). However, this study did not observe a correlation between race and prognosis in ECCC patients. The degree of differentiation was not an independent prognostic factor for ECCC. Therefore, we hypothesized that the prognosis was poor irrespective of the degree of differentiation due to the high invasiveness of ECCC and thus had no significant effect on the prognosis of ECCC patients.

The preferred treatment method to cure ECCC is extensive staging surgery, including total uterine and bilateral adnexectomy, pelvic and para-aortic lymph node dissection, more significant omentum biopsy, and examination of the peritoneal washing fluid (20–22). The advantage of surgery is that the tumor stage is more accurately identified, facilitating the subsequent selection of the appropriate adjuvant treatment. Our results revealed that total hysterectomy was a favorable factor for a good prognosis. The risk of death after total hysterectomy was lower than after partial hysterectomy in both early and advanced stages. Therefore, active surgical treatment was recommended for ECCC patients. Patients who cannot undergo radical surgery should also be treated with tumor-reducing surgery, depending on their physical condition. Adjuvant radiotherapy and chemotherapy are fundamental approaches in treating ECCC. Numerous studies underline that the choice of adjuvant radiotherapy and chemotherapy is associated with the stage of ECCC (23). The adjuvant therapy in patients with early ECCC should be chosen based on prognosis-related factors, such as age and the depth of myometrial invasion. Although our results depicted that radiotherapy and chemotherapy had no role in improving the prognosis of patients with early ECCC, this factor did not hinder patients with early ECCC from benefiting from radiotherapy and chemotherapy. Our results were attributed to the SEER database limitations, which did not allow us to know the adjuvant treatment regimen and course, thus preventing the specific stratification study of the enrolled patients.

The FIGO stage of EC represents the pathological surgical stage, which includes factors related to patient prognoses, such as depth of muscular invasion, nodal metastasis, and distant metastasis. It is the primary tool clinicians use to evaluate the prognosis of EC patients. However, the FIGO stage does not include other factors associated with the survival of patients, such as age, marital status, and treatment methods. At the same time, the nomograms contain the demographic, clinical characteristics, and therapeutic approaches of the ECCC patients. Additionally, the DCA curves applied to ECCC patients established that nomograms had better clinical benefits than traditional FIGO stages in stages I/II and III/IV. Therefore, the nomograms had a significant practical value due to their good accuracy, excellent discrimination ability, and clinical benefits.

Compared to the existing prognostic classification, our predictive model demonstrated several strengths. All the clinical variables included in the survival prediction model were easily accessible. This study enrolled ECCC patients; thus, our nomogram was more applicable to ECCC patients than other classification systems. Moreover, the nomogram intuitively and clearly showed 3-, 5- and 8-year survival rates, which is convenient for clinicians. However, this study had several limitations. Firstly, all the variables selected in our study were clinical characteristics. Several genetic and epigenetic features, including Non-Coding RNAs, identified as predictors of EC patients in previous studies (24, 25), were not included in this study due to the limitations of the SEER database. The absence of these new molecular characteristics deteriorated the practicability of the nomogram model. Secondly, this was a retrospective study; thus, the bias significantly affected the results because the information about the patients was partially missing. For instance, the tumor size of 43.2% of the patients was unknown, which significantly reduced the accuracy of the prediction model. Finally, it was unclear whether the patients received neoadjuvant therapy, and the specific information based on surgery, radiotherapy, chemotherapy, and a potential targeted therapy was unknown.

## Conclusions

Nomograms for predicting 3-, 5-, and 10-year OS in ECCC patients were successfully constructed. Moreover, new risk stratification systems were further built to stratify patients into different risk groups. These predictive models may be valuable tools to aid ECCC management and treatment in clinical practice.

## Data availability statement

The raw data supporting the conclusions of this article will be made available by the authors, without undue reservation.

## Author contributions

ZL, XC, and PC conceived and designed the study. YZ and HZ helped in data collection. PC and YZ performed the analysis and wrote the manuscript. All the authors have reviewed the final draft of the manuscript and approved it for publication.

## Conflict of interest

The authors declare that the research was conducted in the absence of any commercial or financial relationships that could be construed as a potential conflict of interest.

## Publisher’s note

All claims expressed in this article are solely those of the authors and do not necessarily represent those of their affiliated organizations, or those of the publisher, the editors and the reviewers. Any product that may be evaluated in this article, or claim that may be made by its manufacturer, is not guaranteed or endorsed by the publisher.
